# Determinants of quality of life among lung cancer patients: insights from a cross-sectional study

**DOI:** 10.1007/s00406-025-02086-w

**Published:** 2025-08-28

**Authors:** Theresa Halms, Martina Strasser, Alkomiet Hasan, Tobias Rüther, Andrea Rabenstein, Martin Trepel, Stephan Raab, Marcus Gertzen

**Affiliations:** 1 Department of Psychiatry and Psychotherapy, Medical Faculty, University of Augsburg, Geschwister-Schönert Straße 1, 86156 Augsburg, Germany; 2 DZPG (German Center for Mental Health), partner site Munich/Augsburg, Augsburg, Germany; 3 Department of Psychiatry and Psychotherapy, University Hospital, Klinikum der Universität München, Ludwig-Maximilians University Munich, Munich, Germany; 4 Department of Hematology and Oncology, Medical Faculty, University of Augsburg, University Hospital Augsburg, Augsburg, Germany; 5 Comprehensive Cancer Center Augsburg (CCCA), Augsburg, Germany; 6 Department of Thoracic Surgery, University Hospital Augsburg, Augsburg, Germany

**Keywords:** Quality of life, Lung cancer, Smoking, Tobacco, Smoking cessation

## Abstract

**Purpose:**

Lung cancer (LC) is a leading cause of cancer-related mortality, with smoking being a major risk factor. Despite the benefits of smoking cessation, many LC patients continue to smoke, potentially impacting their quality of life (QoL). This study aimed to explore factors influencing QoL among LC patients, with a focus on smoking status and mental health.

**Methods:**

A cross-sectional study was conducted at the University Hospital of Augsburg from December 2021 to December 2023. A total of 56 LC patients were categorized into active smokers (AS), ex-smokers (ES), or never-smokers (NS). Participants completed validated questionnaires assessing QoL, depression, anxiety, stress, borderline personality disorder symptoms, and pain. Statistical analyses, including one-way ANOVA and Pearson’s correlation, were employed to examine group differences and the relationship between QoL and mental health factors.

**Results:**

No significant differences in overall QoL were observed among AS, ES, and NS. However, mental health indicators—including depression, anxiety, stress, and borderline personality disorder symptoms—were significantly negatively correlated with QoL across all groups. Pain was also a key factor affecting QoL. These findings suggest that while smoking cessation is critical for improving prognosis in LC patients, mental health and pain management are more pivotal in determining QoL.

**Conclusion:**

This study emphasizes the need for a holistic approach to LC patient care, addressing both physical and mental health. Future research should focus on longitudinal studies to better understand the long-term effects of smoking and other influencing factors on QoL in this patient population.

**Supplementary Information:**

The online version contains supplementary material available at 10.1007/s00406-025-02086-w.

## Introduction

Lung cancer (LC) is the most frequently diagnosed cancer worldwide, with approximately 2.5 million new cases reported in 2022, representing 12.4% of all cancer diagnoses [1]. The global burden of LC is reflected in the age-standardized rate per 100,000 for incidence by the WHO, with rates reported as 31.9 in the United States, 28.6 in Europe, and 28.1 in Germany [2]. It also remains the leading cause of cancer-related mortality globally, accounting for 1.8 million deaths, or 18.7% of all cancer fatalities in the same year [1]. Correspondingly, the mortality rates per 100,000 are 16.6, 21.7, and 16.8 per 100,000 in the United States, Europe, and Germany, respectively [2]. Due to the often delayed diagnosis, the prognosis for LC is particularly poor, with a 5-year survival rate estimated at around 25% for women and 19% for men [3], though some studies suggest it may be as low as 15% [4]. Patients with advanced LC endure a range of debilitating symptoms, such as dyspnea, chronic cough, and chest pain, which are often exacerbated by the side effects of treatments like chemotherapy, radiotherapy, and surgery, including nausea, fatigue, hair thinning, loss of appetite, and gastrointestinal issues [5]. These symptoms and side effects severely impair patients’ quality of life (QoL), leading to significant physical, emotional, and functional challenges. As a result, the QoL reported by LC patients is significantly lower compared to both the healthy population and patients with other diseases [4].

Additionally, mental health problems such as depression and anxiety are more prevalent in this patient group [6]. One meta-analysis of 183 studies and a total of 182,521 cancer patients found that LC patients had the second highest depression rates compared to patients with other cancer types [7]. The results showed that 31% of LC patients met the criteria for depression [7]. Further, another meta-analysis investigating the prevalence of anxiety symptoms among cancer patients demonstrated an anxiety prevalence of 26% among LC patients [8]. Additionally, a cross-sectional study including 149 LC patients revealed an association between increased levels of depression, anxiety, LC stigma and lower health-related quality of life [9]. These results were confirmed by another study including 204 LC patients, which showed that LC patients with depressive symptoms reported a significantly lower QoL than those without depression [10]. Further evidence suggests that certain characteristics—such as younger age, female gender, and current cigarette smoking—put lung cancer patients at a higher risk for emotional problems [11].

Smoking is the major risk factor for LC, with nearly 90% of LC patients having a history of smoking. A substantial proportion of patients continue to smoke during the diagnostic process, with estimates ranging from 23 to 60% [12, 13]. Even after a lung cancer diagnosis, about 20–30% of patients persist in smoking [14–17]. Regardless of the type of lung cancer and the stage of the disease, the continuation of smoking post-diagnosis is associated with a host of negative outcomes, including increased treatment toxicity, higher risk of treatment failure, greater likelihood of developing second primary tumors, and reduced survival rates [18]. While smoking cessation is known to improve treatment outcomes and overall survival in LC patients [19], maintaining long-term abstinence remains a significant challenge. The diagnosis of LC often serves as a “wake-up call”, heightening patient awareness of the health risks associated with smoking and providing a crucial opportunity (“teachable moment”) for healthcare professionals to discuss lifestyle changes [20]. While many patients are initially motivated to quit smoking following their diagnosis, long-term smoking abstinence rates tend to decline over time, with success rates ranging between 40% and 87% [21]. Relapses are commonly driven by factors such as nicotine withdrawal, pain, fatigue, nausea, depression, and anxiety [18].

Moreover, the impact of smoking on QoL in LC patients is an area of growing interest. It is well-documented that the QoL of smokers is generally lower compared to non-smokers, yet research on LC patients has yielded inconsistent results regarding the relationship between smoking status and QoL A recently published systematic review of 23 included studies on the impact of smoking status on QoL in LC patients showed mixed results, with a general trend towards lower QoL among LC patients who smoke [22].

Given the complex and multifaceted factors affecting QoL among LC patients, this study aims to comprehensively investigate various influences on QoL, with particular emphasis on the potential associations between QoL, mental health, and smoking status. By exploring psychological conditions such as depression and anxiety alongside smoking behavior, the study seeks to identify critical determinants affecting patient well-being. Findings from this analysis will inform strategies to enhance patient management and develop targeted supportive interventions to improve QoL for individuals diagnosed with LC.

## Methods

### Study design and setting

We conducted a descriptive, observational, cross-sectional study. Patients with a diagnosis of LC were recruited from the Comprehensive Cancer Center Augsburg (CCCA) at the University Hospital Augsburg, Germany, between December 2021 and December 2023. This study was registered with the number DRKS00026813 at the German Register of Clinical Studies (DRKS).

### Procedure and eligibility criteria

Health professionals identified potential participants during routine clinic contacts and referred them to the study team. Eligible participants had to meet the following criteria: (a) aged 18 years or older, (b) be a patient at the Comprehensive Cancer Center Augsburg (CCCA), (c) have a diagnosis of LC, (d) be able to understand the study information, (e) be deemed physically and psychologically fit to participate by a member of the study team, and (f) be not suicidal. Patients who did not meet any of these criteria were excluded. Patients suitable for participation were approached by study members who provided verbal and written information about the study. Participants who smoked regularly (at least one cigarette per day, five cigarettes per week or one pack per month) were categorized as active smokers, participants who smoked regularly in the past but quit smoking prior to participation in the survey were categorized as former smokers and patients who had never smoked regularly were categorized as never smokers. Smoking patients underwent a thorough assessment of their current smoking behavior, including the self-assessment Fagerström Test to classify the degree of dependence. Participants who consented to the study were given questionnaires to complete either in the clinic or at home. They were informed that they could contact the study team at any time with questions or concerns and that they could withdraw from the study at any time without providing a reason or facing any disadvantages. If actively smoking patients expressed a desire to quit, we offered tobacco cessation counseling. The study was approved by the ethics committee of the Medical Faculty of the Ludwig-Maximilians-University (LMU) Munich, Germany (Ref. 21–0583) and was performed in accordance with the ethical standards as defined in the Declaration of Helsinki. The participants provided their written informed consent to participate in this study.

### Outcome measures

Participants were provided with validated German-language self-assessment scales to measure various psychological and physical parameters. These included the Beck Depression Inventory-II (BDI-II) for depressive symptoms, the Depression Anxiety Stress Scales (DASS) for anxiety and stress symptoms, the McGill Pain Questionnaire-Short Form (SF-MPQ) for pain, the Borderline Symptom List-23 (BSL-23) for personality traits, the Test of Self-Conscious Affects (TOSCA) for feelings of shame and guilt, and the McGill Quality of Life Questionnaire-Revised (MQOL) for quality of life. Depression was assessed both as a subdomain of the Depression Anxiety Stress Scales (DASS-21) and via the Beck Depression Inventory-II (BDI-II), as the latter is focused on depression and includes a wider range of items. Additionally, participants received a questionnaire about sociodemographic information, other potential substance use disorders (e.g., cannabis or alcohol), somatic comorbidities, current medication, current smoking status, as well as exposure duration and dose. We assessed the “gender” of participants, referring to self-reported data provided by participants. This approach allowed individuals to define their gender identity based on their own perceptions and experiences, without making distinctions between biological sex and gender. Smoking participants were also queried about their reasons for wanting to quit and their confidence in doing so, using the Self-Efficacy Scale for Smoking Cessation (SER).

### Statistical analysis

Analyses were conducted using SPSS (Version 29.0). Descriptive statistics were first calculated to summarize participant characteristics. Due to the number of missing values for individual items within the questionnaires, likely attributable to the participants’ poor physical condition and the comprehensiveness of the questionnaire for a non-primarily mentally affected population, individual mean scores of complete items instead of sum scores were calculated for all scales. This approach allowed for the inclusion of participants with incomplete data while maintaining the integrity of the overall score interpretation. Further, the evaluation of the TOSCA questionnaire was not feasible due to an excessive number of missing values. Consequently, this scale was excluded from further analyses. Additionally, ANOVA was used to examine differences in psychosocial and physical health indicators across groups. Exploratory Pearson’s correlation coefficients were calculated to explore the relationships between QoL scores and psychosocial and physical health indicators. To control for multiple testing in a total of 30 correlations, significance level for Pearson’s correlation coefficients was Bonferroni-adjusted to α = 0.05/30 = 0.0017.

## Results

### Demographics

A total of 266 patients were screened for eligibility, 86 of whom were found not to have LC. A further 58 patients declined to participate in the study or underwent only brief hospital stays, thereby preventing completion of the questionnaire. Additionally, 49 patients were hospitalized multiple times during the study period and were therefore repeatedly invited to participate. Another 17 patients agreed to participate in the study but provided no or insufficient answers. Consequently, a total of 56 patients provided sufficient data for further analysis. Factors contributing to the lower number of study participants include the restrictions imposed by the COVID-19 pandemic, in particular the reduced occupancy of beds, an overestimation of the number of new LC admissions per week as well as the critical state of health of numerous patients, which prevented them from completing the questionnaire. Cancer stages were distributed as follows: 15.38% stage I, 12.82% stage II, 15.38% stage III, and 56.41% stage IV. An additional 30.36% patients did not report their cancer stage. Most participants (59%) were former smokers, with 27% current smokers and 14% never-smokers. No significant pack-year differences were found between active and ex-smokers (Z = − 0.46, *p* = .649). Over 70% of participants lived with others, and 64.29% had attended at least one social gathering in the past week. The respondents exhibited considerable variation in educational attainment, with 53.57% having obtained qualifications at the lower secondary school level. For a detailed overview of the descriptive data, please refer to Table 1.

**Table 1:** Sociodemographic characteristics of study sample

	Total *N* = 56		
	n	M	SD
*Age (in years)*	55	63.71	9.29
	n	M	SD
*Pack years*	38	48.28	41.09
	**n**	**%**	
*Smoking status*			
Active smokers (AS)	15	26.79	
Ex-smokers (ES)	33	58,93	
Never-smokers (NS)	8	14,29	
*Cancer stage*			
I	6	15.38	
II	5	12.82	
III	6	15.38	
IV	22	56.41	
Not reported	17	30.36	
*Gender*			
Female	30	53.57	
Male	26	46.43	
*Psychiatric/psychotherapeutic treatment*			
Currently in treatment	9	16.07	
Previously in treatment	19	33.93	
*Living situation*			
Alone	16	28.57	
With partner/family/friends	40	71.43	
*Social gatherings within the past week*			
None	20	35.71	
At least one	36	64.29	
*Education*			
No qualifications	4	7.14	
Special school qualification	0	0.00	
Lower secondary school qualification	30	53.57	
Secondary school qualification	14	25.00	
Higher school qualification	4	7.14	
University degree	4	7.14	
*Employment status*			
In training	0	0.00	
Working	4	7.14	
On sick leave	12	21.43	
Unemployed	2	3.57	
Retired early	6	10.71	
Retired	27	48.21	
Other	5	8.93	

*N* Number of participants (group size), *LC* Lung cancer, *M* Mean, *SD* Standard deviation

### Associations between smoking status and qol, mental health indicators and pain

Mean overall QoL scores were 7.32 for active smokers, 6.57 for ex-smokers, and 6.74 for never-smokers, with no significant differences (F_(2, 52)_ = 0.72, *p* = .493). Physical QoL scores were also similar, with no significant differences (F_(2, 52)_ = 0.063, *p* = .536). Psychological, existential and social QoL showed no significant group differences, though active smokers had slightly higher social QoL (F_(2, 52)_ = 3.00, *p* = .058). Further, ANOVA revealed no significant group differences in mental health indicators or pain (all *p* > .05). Depression, anxiety, stress, borderline personality disorder (BPD) and pain scores were comparable across groups. For a detailed overview of the descriptive data and test statistics, please refer to Table 2.

With regard to the potential impact of psychiatric treatment and cancer stage on patients’ state of mental health, our analyses revealed no significant group differences between individuals with and without current or previous psychiatric/psychotherapeutic treatment in any mental health indicators or pain (all *p* ≥ .114). Similarly, Welch’s ANOVAs revealed no significant differences in psychological symptom scores across cancer stages (Stage I–IV) for any variable (all *p* ≥ .414). See Supplementary Tables 1 and 2 for descriptive data and test statistics.

**Table 2:** Results of the comparison between active, ex- and non-smokers for QoL and mental health indicators/pain

	AS			ES			NS				df		
McGill Quality of Life Questionnaire	N	M	SD	N	M	SD	N	M	SD	F	BG	WG	p
Overall QoL	14	7.32	1.37	33	6.57	2.12	8	6.74	2.08	0.717	2	52	0.493
Physical QoL	14	5.99	1.94	33	6.00	2.70	8	4.88	3.18	0.632	2	52	0.536
Psychological QoL	14	7.23	2.40	33	6.42	3.24	8	8.13	2.20	1.240	2	52	0.298
Existential QoL	14	7.34	1.46	33	7.01	1.95	8	6.84	2.24	0.218	2	52	0.805
Social QoL	14	9.14	1.63	33	7.18	2.93	8	7.13	2.51	3.002	2	52	0.058
*Borderline symptom (BSL-23)*	15	0.39	0.43	32	0.47	0.49	8	0.32	0.55	0.363	2	52	0.698
*Depression (DASS-21)*	15	0.74	0.74	33	0.90	0.80	8	0.46	0.81	1.063	2	53	0.353
*Anxiety (DASS-21)*	15	0.60	0.54	33	0.72	0.68	8	0.57	0.80	0.246	2	53	0.783
*Stress (DASS-21)*	15	0.99	0.80	33	0.98	0.84	8	0.57	0.61	0.901	2	53	0.412
*Depression (BDI)*	15	0.53	0.38	33	0.56	0.46	8	0.38	0.34	0.585	2	53	0.561
*Pain (McGill Pain Questionnaire SF)*	13	0.57	0.55	32	0.58	0.79	7	0.75	0.76	0.170	2	49	0.844

*N* number of participants (group size), *QoL* Quality of Life, *AS* Active smokers, *ES* Ex-smokers, *NS* non-smokers, *M* mean, *SD* Standard deviation, *F* F-statistics, *BG* Between groups, *WG* Within groups, *df* Degrees of freedom p: p-value.

### QoL and mental health indicators/pain

**Fig. 1:**
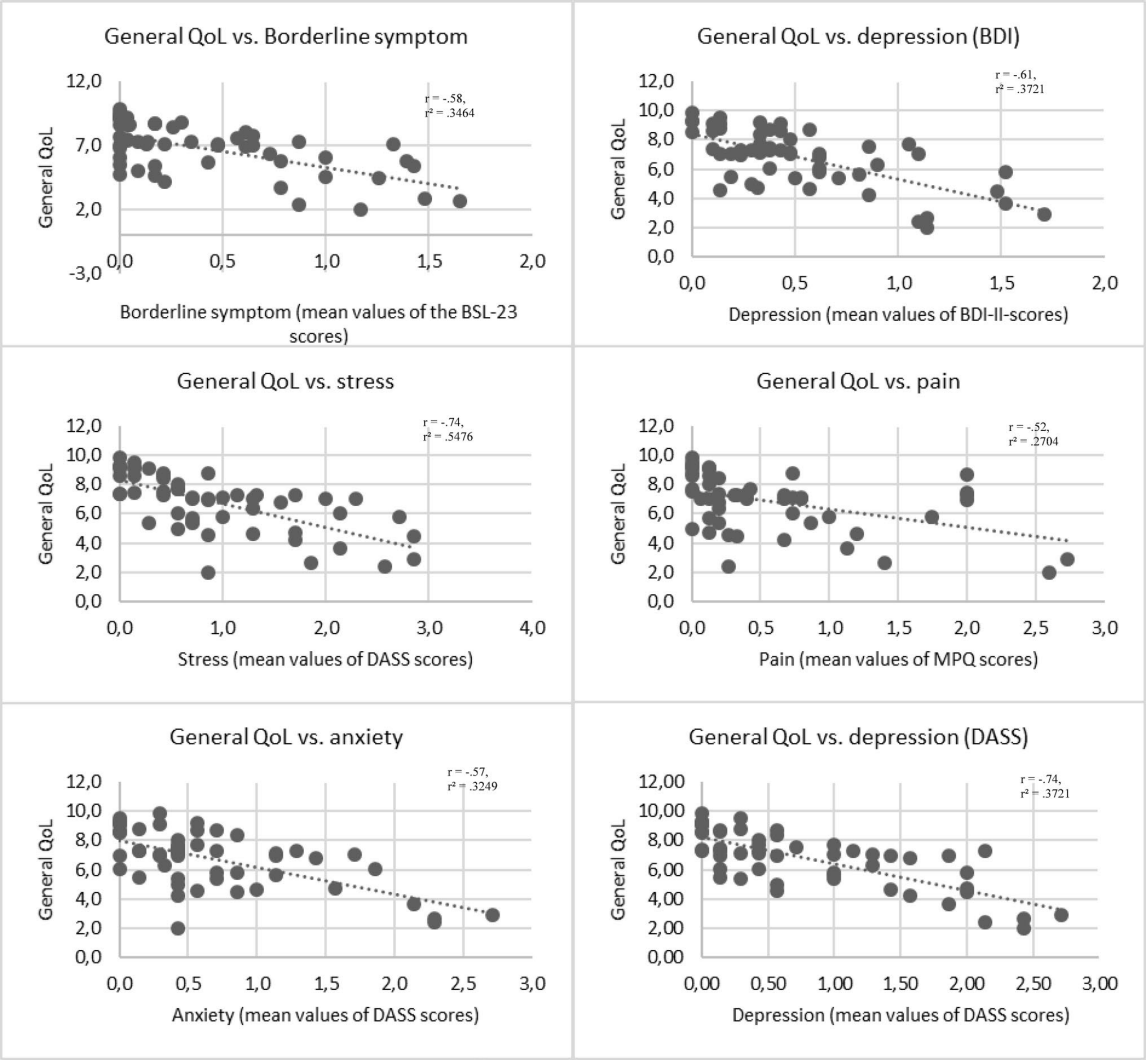
Scatter plots of the correlations between general QoL and psychiatric disorders

Pearson’s correlations showed significant negative associations between overall QoL and mental health indicators (see Fig. 1), including BPD (*r* = − .58, *p* < .001), depression (DASS-21, *r* = − .74, *p* < .001), anxiety (*r* = − .57, *p* < .001), stress (*r* = − .74, *p* < .001), depression (BDI-II, *r* = − .61, *p* < .001) and pain (*r* = − .52, *p* < .001). Similar negative correlations were found for physical, psychological, existential and social QoL. For a detailed overview of the descriptive data and test statistics, please refer to Table 3.

**Table 3:** Results of the correlations between quality of life and psychiatric disorders

	Borderline symptom (BSL-23)			Depression (DASS-21)			Anxiety (DASS-21)			Stress (DASS-21)			Depression (BDI-II)			Pain (MPQ)		
	N	r	p	N	r	p	N	r	p	N	r	P	N	r	p	N	r	p
Overall QoL	54	− 0.58	< 0.001*	55	− 0.74	< 0.001*	55	− 0.57	< 0.001*	55	− 0.74	< 0.001*	55	− 0.61	< 0.001*	52	− 0.52	< 0.001*
Physical QoL	54	− 0.47	< 0.001*	55	− 0.53	< 0.001*	55	− 0.47	< 0.001*	55	− 0.53	< 0.001*	55	− 0.48	< 0.001*	52	− 0.58	< 0.001*
Psychological QoL	54	− 0.50	< 0.001*	55	− 0.76	< 0.001*	55	− 0.59	< 0.001*	55	− 0.80	< 0.001*	55	− 0.62	< 0.001*	52	− 0.45	< 0.001*
Existential QoL	54	− 0.48	< 0.001*	55	− 0.57	< 0.001*	55	− 0.42	0.001*	55	− 0.58	< 0.001*	55	− 0.47	< 0.001*	52	− 0.42	0.002
Social QoL	54	− 0.45	< 0.001*	55	− 0.41	0.002	55	− 0.25	0.071	55	− 0.33	0.014	55	− 0.40	0.002	52	− 0.24	0.084

*N* number of participants (group size), *QoL* Quality of Life, *r* r-statistic, *p* p-value. *p < .05, Bonferroni-adjusted to α = 0.0017

## Discussion

The objective of the present study was to identify and analyze factors that influence the QoL among LC patients. The findings offer valuable insights into the impact of smoking status and psychosocial aspects on the QoL of this specific population. In general, indicators of psychosocial health were found to be significantly associated with QoL. In particular, the findings indicated that an increase in BPD symptoms, depression, anxiety, stress, and pain levels were associated with a decline in QoL. However, the results demonstrated that smoking status had no notable effect on the QoL of LC patients.

Our analysis revealed no significant differences in overall QoL or its subdomains (physical, psychological, existential, and social) among active, ex-, and never-smokers. Our findings are partially consistent with the results of a recently published systematic review examining the impact of smoking on QoL in LC patients [22]. The review demonstrated a negative impact of smoking on QoL in a total of 12 of the 23 studies included. Conversely, 10 studies found no differences between smoking groups, one reported better QoL in patients who were active smokers, and three studies indicated trends toward a negative impact of smoking on QoL [22]. In contrast, findings among a broad sample of healthy subjects and patients with different diagnoses have contradicted the results of the present study. One meta-analysis comprising 44 studies demonstrated a negative correlation between smoking and QoL, as well as a significant improvement in QoL following smoking cessation [23]. A similar result was observed in a study of nearly 100 patients with head and neck cancer, which confirmed a negative correlation between smoking and QoL [24]. Conversely, a longitudinal study conducted over a twelve-month period investigating the impact of smoking cessation on QoL in patients with coronary heart disease revealed no significant differences between patients who quit smoking and those who continued to smoke [25]. Similarly, a systematic review of 1,288 bladder cancer survivors revealed no significant association between smoking status and overall QoL [26]. In light of the conflicting evidence regarding the effect of smoking on the QoL of patients with diverse diagnoses, it can be postulated that the impact of smoking on QoL may not be uniform across all populations. In particular, it appears that no definitive conclusion can be drawn regarding the relationship between smoking status and QoL among LC patients. This may be due to the influence of other factors that have a more pronounced impact on the QoL of this specific population. Moreover, the diversity of instruments utilized to assess QoL must be considered. These instruments vary in their focus on specific subdomains of QoL, and in their incorporation of disease-specific parameters. Consequently, the use of less differentiated scales for the assessment QoL may yield disparate results. In the context of the present study, no significant associations were identified between smoking and QoL for any of the subdomains considered in the McGill QoL Scale. The aforementioned findings, both past and present, illustrate the intricate nature of the factors influencing QoL. They suggest that factors beyond smoking status may play a more pivotal role in determining QoL among LC patients.

In this regard, the results of our study indicate that psychosocial health indicators in particular substantially contribute to the QoL of LC patients. Accordingly, negative associations between depression, anxiety, stress and BPD and general QoL as well as all subdomains were demonstrated among the LC patients included in the present study. Similarly, the adverse effect of pain on the QoL of our study sample was revealed. The negative impact of poor mental health and pain on QoL among LC patients has previously been confirmed in several studies [27, 28], further strengthening the results of our study. Moreover, the severe impact of depressive symptoms and anxiety has been demonstrated in another cross-sectional study among LC patients. The results indicated that depression and anxiety were both associated with decreased QoL, while depression was further associated with poor treatment adherence and prognosis [29]. In addition, the negative correlation between mental burden and QoL has previously been established among patients suffering from other cancers. For instance, one cross-sectional study including, amongst others, breast, lung and colorectal cancer patients revealed that psychological distress had a significant negative impact on the QoL of cancer patients [30]. Further, one meta-analysis including 64 articles and a total of 28,423 patients investigating the QoL and its determinants among cancer survivors demonstrated large, negative effect sizes for depression and anxiety on QoL [31]. Another study on the influence of anxiety and depression on QoL in a total sample of 405 cancer patients concluded that both depression and anxiety were associated significantly with the mental subdomain of QoL as well as somatic symptom burden [32]. However, only depression was found to have a significant effect on other domains and overall QoL [32]. Overall, the uniform impact of psychosocial health indicators on QoL of cancer patients implies that the psychological and physical burden of LC may be equally experienced across different smoking histories. This uniformity underscores the pervasive impact of LC on patients’ well-being, potentially transcending the influence of smoking.

Another aspect requiring consideration when investigating QoL and its determinants is the complexity of the multifaceted concept of QoL. Accordingly, one systematic review on self-administered questionnaires used among the group of cancer patients to assess QoL identified a total of 39 different instruments [33]. The review further revealed that the majority of the included instruments had been inadequately tested regarding construct validity and reliability, which in turn impedes the selection of a suitable instrument for measuring the QoL of cancer patients [33]. Furthermore, when evaluating QoL using self-administered instruments, the subjectivity and individuality of patients’ perceived QoL as well as fluctuations in the assessment of QoL at different points in time need to be considered. Additionally, patients with chronic illnesses may experience adaption to certain circumstances and physical limitations, which may impact their self-reported QoL. Thus, while QoL serves as a significant endpoint in health research and is crucial for making informed medical decisions [34], its adequate assessment poses certain challenges.

## Limitations

Several limitations must be acknowledged. The cross-sectional study design limits the ability to infer causality of the obtained results. Thus, our findings are limited to a single snapshot in time, which may not accurately reflect changes over time regarding the long-term impact of smoking on QoL. While a longitudinal design may be more effective in understanding how these variables interact over time, LC patients represent a distinct patient group that can be difficult to reach due to the often rapid progression of the disease and its symptoms or poor compliance. Another limitation is the lack of a non-cancer comparison group, which limits conclusions about whether the findings are specific to cancer patients. Moreover, while the results of the exploratory correlations offer valuable insights, the identified associations require confirmation in future studies and should thus be interpreted with caution. Further, the assessment of self-reported data is another limitation, as it can introduce bias due to under- or overreporting of smoking habits, psychological burden or QoL due to social desirability or recall inaccuracies. Objective measures, such as biochemical verification of smoking status, could provide more reliable data. Additionally, the sample size, particularly within subgroups, may limit the generalizability of the findings.

## Conclusion

Overall, the results of our study highlight the complexity of QoL determinants and suggest that factors beyond smoking status may play more pivotal roles in influencing QoL among LC patients. Thus, the need for a holistic approach to LC patient care, focusing not only on smoking cessation but also on comprehensive management of mental and physical health becomes apparent. Nevertheless, due to the proven positive impact of smoking cessation on prognostic outcomes, treatment efficacy, recurrence and mortality [19, 35], smoking cessation remains a crucial recommendation for all patients, irrespective of its impact on QoL. Multidisciplinary interventions addressing mental health, social support, and symptom management are essential. Future research should focus on long-term effects of smoking on QoL among this particular group of patients and consider potential confounding variables, which may in turn provide a more nuanced understanding of factors impacting QoL.

## Supplementary Information

Below is the link to the electronic supplementary material.

Supplementary Material 1

## Data Availability

The datasets used and/or analyzed during the current study are available from the corresponding author on reasonable request.
