# Supplementary material for: Determinants of quality of life among lung cancer patients: insights from a cross-sectional study
Source: Eur Arch Psychiatry Clin Neurosci. 2025 Aug 28;276(1):369–78. doi: 10.1007/s00406-025-02086-w (PMC12904944; doi:10.1007/s00406-025-02086-w)
Supplement: Supplementary file 1 — Supplementary Material 1 [file 406_2025_2086_MOESM1_ESM.docx]

**Supplementary Table 1: Results of the comparison between patients receiving/not receiving current or previous psychiatric/psychotherapeutic treatment for secondary outcomes**

|  | **CPT** | | | **NPT** | | | **CPT vs. NPT** | | | **PPT** | | | **NPPT** | | | **PPT vs. NPPT** | | |
| --- | --- | --- | --- | --- | --- | --- | --- | --- | --- | --- | --- | --- | --- | --- | --- | --- | --- | --- |
|  | **N** | **M** | **SD** | **N** | **M** | **SD** | **U** | **Z** | **p** | **N** | **M** | **SD** | **N** | **M** | **SD** | **U** | **Z** | **p** |
| **Borderline symptom (BSL-23)** | 9 | .47 | .47 | 47 | .41 | .48 | 183.00 | -.64 | .590 | 19 | .48 | .52 | 36 | .39 | .46 | 304.50 | -.81 | .509 |
| **Depression (DASS-21)** | 9 | .83 | .79 | 47 | .79 | .80 | 196.50 | -.33 | .745 | 19 | .98 | .87 | 37 | .71 | .74 | 289.00 | -1.08 | .280 |
| **Anxiety (DASS-21)** | 9 | .50 | .37 | 47 | .67 | .70 | 225.50 | .31 | .761 | 19 | .72 | .66 | 37 | .63 | .66 | 303.00 | -.84 | .402 |
| **Stress (DASS-21)** | 9 | .98 | .84 | 47 | .91 | .80 | 199.00 | -.28 | .788 | 19 | 1.11 | .90 | 37 | .83 | .74 | 286.00 | -1.13 | .259 |
| **Depression (BDI)** | 9 | .57 | .44 | 47 | .52 | .42 | 192.00 | -.44 | .671 | 19 | .62 | .46 | 37 | .48 | .40 | 290.50 | -1.06 | .294 |
| **Pain (McGill Pain Questionnaire SF)** | 8 | .31 | .35 | 44 | .66 | .76 | 221.00 | .21 | .256 | 17 | .70 | .63 | 35 | .56 | .76 | 216.50 | -1.58 | .114 |

N: number of participants (group size), CPT: Currently in psychiatric/psychotherapeutic treatment, NPT: Not currently in psychiatric/psychotherapeutic treatment, PPT: Previously in psychiatric/psychotherapeutic treatment, NPPT: Never in psychiatric/psychotherapeutic treatment, M: mean, SD: Standard deviation, U: Mann-Whitney-U statistic, Z: Z-value, p: p-value.

**Supplementary Table 2:** **Results of the comparison between patients with cancer stages I-IV for secondary outcomes**

|  | **LC Stage I** | | | **LC Stage II** | | | **LC Stage III** | | | **LC Stage IV** | | |  | **df** | |  |
| --- | --- | --- | --- | --- | --- | --- | --- | --- | --- | --- | --- | --- | --- | --- | --- | --- |
|  | **N** | **M** | **SD** | **N** | **M** | **SD** | **N** | **M** | **SD** | **N** | **M** | **SD** | **F** | **BG** | **WG** | **p** |
| **Borderline symptom (BSL-23)** | 6 | .68 | .61 | 5 | .45 | .40 | 6 | .38 | .54 | 21 | .38 | .51 | .358 | 3 | 10.55 | .784 |
| **Depression (DASS-21)** | 6 | .88 | .88 | 5 | .74 | .94 | 6 | .64 | .64 | 22 | .88 | .80 | .193 | 3 | 10.25 | .899 |
| **Anxiety (DASS-21)** | 6 | .76 | .59 | 5 | .58 | .97 | 6 | .45 | .38 | 22 | .81 | .73 | .856 | 3 | 10.97 | .492 |
| **Stress (DASS-21)** | 6 | 1.52 | 1.17 | 5 | 1.03 | .99 | 6 | .67 | .60 | 22 | .93 | .75 | .794 | 3 | 9.88 | .525 |
| **Depression (BDI)** | 6 | .75 | .59 | 5 | .55 | .43 | 6 | .49 | .37 | 22 | .52 | .40 | .271 | 3 | 9.93 | .845 |
| **Pain (McGill Pain Questionnaire SF)** | 6 | 1.01 | .84 | 5 | .52 | .83 | 5 | .35 | .33 | 20 | .57 | .65 | 1.044 | 3 | 10.21 | .414 |

N: number of participants (group size), LC: Lung cancer, M: mean, SD: Standard deviation, F: Welch’s F-statistics, BG: Between groups, WG: Welch-corrected df within groups, df: Degrees of freedom p: p-value.
